# Managing Ectopic Pregnancies by Targeting Chorionic Villi with a Transvaginal Injection of Ethanol into the Lacunar Space

**DOI:** 10.3390/biomedicines8070202

**Published:** 2020-07-09

**Authors:** Hisao Osada, Shokichi Teramoto, Hirotsune Kaijima, Tomoya Segawa, Masaji Nagaishi, Makio Shozu, Keiichi Kato

**Affiliations:** 1 Natural ART Clinic Nihombashi, 8F Tokyo Nihombashi Tower, 2-7-1 Nihombashi, Chuo-ku, Tokyo 103-6008, Japan; s.teramotofamily@gmail.com; 2 Minatomirai Yume Clinic, 2F MM Park Building, 3-6-3 Minatomirai, Nishi-ku, Yokohama-shi, Kanagawa 220-0012, Japan; h-kaijima@mm-yumeclinic.com; 3 Shimbashi Yume Clinic, EXCEL Shimbashi, 2-5-1, Shimbashi, Minato-ku, Tokyo 105-0004, Japan; t-segawa@yumeclinic.net; 4 Department of Obstetrics and Gynecology, School of Medicine, Nihon University, 30-1 Ohyaguchi Kamimachi, Itabashi-ku, Tokyo 173-8610, Japan; nmasaji@gc4.so-net.ne.jp; 5 Department of Reproductive Medicine, Graduate School of Medicine, Chiba University, 1-8-1 Inohana, Chuo-ku, Chiba Prefecture, Chiba 260-0876, Japan; shozu@faculty.chiba-u.jp; 6 Kato Ladies Clinic, Westgate Shinjuku Building, 7-20-3, Nishi-Shinjuku, Shinjuku-ku, Tokyo 160-0023, Japan; k-kato@towako.net

**Keywords:** chorionic villi-targeted therapy, ectopic pregnancies, absolute ethanol, nonsurgical management, transvaginal ultrasonography, methotrexate

## Abstract

Methotrexate has been the main mode of non-surgical treatment for ectopic pregnancies. However, we have developed an easier, repeatable method that can be applied even to patients with a high beta-human chorionic gonadotropin (β-hCG) level and/or positive fetal heartbeat, by targeting chorionic villi with a transvaginal injection of absolute ethanol (AE) into the lacunar space (intervillous space). The efficacy and safety of this method were examined in 242 cases of ectopic pregnancy, including 103 with positive fetal heartbeat. Serum β-hCG level was measured at frequent intervals, and transvaginal ultrasonography was performed to observe the gestational sac and hyperechoic inner ring. Of the 242 patients, 222 (91.7%) were successfully treated. The average number of AE injection(s) required was 1.6 (range: 1–5), and the average dose was 3.2 mL. After the treatment, many of the patients tried to conceive again, and 63 of the traceable 145 patients (43.4%), who had fallopian tube pregnancy, and 7 of the traceable 12 patients (58.3%), who had cervical or cesarean scar pregnancies, successfully conceived and delivered babies with no observed side effects. Therefore, this method could be an effective treatment for ectopic pregnancy with the potential to replace conventional surgical interventions and medical treatment using methotrexate.

## 1. Introduction

Ectopic pregnancies (EP), which account for approximately 2% of all pregnancies, can seriously compromise a patient’s health and future fertility [1,2]. Most EPs are tubal, although a few have other causes as well, for example, cervical pregnancy (CP) and cesarean section scar pregnancy (CSSP). CP and CSSP are rare, with reported incidences of 1 in 1000 to 95,000 pregnancies [3], and 1 in approximately 2000 in patients with previous CSSP [4], respectively. However, they are among the most difficult conditions to treat, and hysterectomy or uterine artery embolization is often necessary.

For nearly four decades, methotrexate (MTX) has been recognized as an effective drug for treating EPs and has been used as a non-surgical medical treatment in various protocols, including both in situ injection and systemic administration [5,6]; however, a consensus regarding the optimal method has not yet been reached. Local injection of MTX is an effective technique for preserving fertility in women with lower uterine EP, and Yamaguchi et al. [6] reported a success rate of 100% in this regard.

However, the challenge with MTX is that its guidelines are not applicable for patients with high beta-human chorionic gonadotropin (β-HCG) or with fetal heartbeat (FHB) [7,8], and that it takes 5–7 days from initial administration to show any effect. Moreover, it takes as long as 2–3 months for the serum β-HCG level to become negative [6].

Meanwhile, local injection of absolute ethanol (AE) has shown advantages over conventional MTX-based local injection therapy in several respects. First, this method is not bound by any of the indication restrictions described in the MTX treatment guidelines. AE is not toxic, and may be repeatedly administered as required. Moreover, the pharmacological effect of AE is immediate, making the AE injection method ideal for treating such high-risk conditions as ectopic pregnancies. Based on our previous experience in using AE for tubal pregnancies [9,10], we had improved this method and named it “trophoblast-target therapy” (TTT) in the previous paper [11]. In this therapy, AE was injected into the lacunar space, rather than into the gestational sac (GS). However, we have now renamed it as “chorionic villi-targeted therapy with absolute ethanol” (CV-T therapy with AE) to be more precise, since AE is targeted to the syncytiotrophoblast, the outer layer of chorionic villi, which facilitates an exchange of material between the mother and the embryo. Since lacunar spaces surround the villi, injecting AE into this space could directly affect the syncytiotrophoblast. This study aimed to report the efficacy and safety of the method using 242 patients.

## 2. Materials and Methods

In this study, we retrospectively evaluated a consecutive series of 242 women with EP, including 16 with CP and 3 with CSSP, 103 (42.5%) of them being FHB-positive, who were treated at Natural ART Clinic Nihombashi, Kato Ladies Clinic, Shinjuku ART Clinic, Nihon University Hospital, and Shimbashi Yume Clinic, between April 2006 and December 2019, with CV-T therapy using AE. All patients consented to undergo this procedure with the understanding that it was not standard care. The study protocol was approved by the Institutional Review Board of Kato Ladies Clinic (No. 17-11; 1 October 2017). Informed consent was obtained from all patients. The diagnosis was made based on each patient’s medical history, clinical examination, serum β-hCG level, findings of TVU and, if necessary, by magnetic resonance imaging (MRI). To assess embryonic activity, the presence of FHB was monitored using transvaginal ultrasound Doppler.

The following diagnostic criteria were applied and, in case of CP and CSSP, criteria reported in the literature [12,13,14,15] were applied as well, namely: (1) no identifiable intrauterine pregnancy was present despite a normal increase in maternal serum β-hCG, detected after 3 w 5 d of GA; (2) GS containing the viable embryo was located below the level of internal orifice of the cervix uteri within the cervical tissue, and the cervix became hour-glass- or barrel-shaped as the GS expanded (for CP); (3) the sliding sign of miscarriage was absent (in a miscarriage, the GS will slide into the cervical canal when pressure is applied to the cervix using a probe) [16]; and (4) the uterine cavity and cervical canal were empty, GS was present in the anterior part of the uterine isthmus, and myometrial tissue between the bladder and GS was absent or defective (for CSSP).

Serum β-hCG level was measured by the enzyme immunoassay method (AIA-1800; Tosoh, Tokyo, Japan). Monitoring of the β-hCG level, after embryo transfer, began at 3 w 5 d of gestational age (GA). GS and FHB were monitored from 5 w 0 d and 6 w 0 d of GA, respectively.

All 242 patients, including those who were cared for in the outpatient setting, received AE injections in the operating room. Under exceptional circumstances, such as when the crown-to-rump length was greater than usual in 7 weeks and in presence of bleeding, the patient remained in the hospital. Other patients, who had come from far away, preferred to be hospitalized. The local AE injection was performed following the principle of no anesthesia under ultrasound guidance using a 23-G needle. If the patient desired anesthesia, local anesthesia of the uterine wall was performed using 1% Lidocaine^R^ or 0.5% Marcain^R^.

### 2.1. Identification of the Lacunar Space by TVU and Local Injection of AE

The lacunar space occupies the hyperechoic ring (decidua capsularis) created around the GS by chorionic response [17,18]. In the CV-T therapy, 100% pure AE (Anhydrous Ethanol Injection; Pfizer, Tokyo, Japan) was injected into the lacunar space, rather than in the GS, under high-intensity imaging TVU guidance, using a 23-G Cathelin needle (Kitazato Medical, Tokyo, Japan).

The average injected dose of AE was 3.2 mL (range: 0.3–53.0 mL), depending on the thickness of the hyperechoic ring and dispersion of the liquid. When GS was small and AE could be smoothly injected, only a minimal amount was required, starting at 0.3 mL. However, when the GS was large, or AE injection was difficult, a higher dose was required.

Since 1 mL of AE was injected into the tissues over 30 to 40 pushes (0.025–0.03 mL per push), AE did not enter directly into the vein, and thus did not circulate in the systemic blood flow. Occasionally, the white ring was not visible clearly. In such cases, transvaginal ultrasound Doppler image was used to detect the presence of blood flow where the white ring should have been, and AE was injected into the site. The cases in which the white ring was not clearly visible tended to require multiple treatments owing to uncertainty regarding the exact site of injection.

### 2.2. Evaluation of the Effect of CV-T Therapy with AE

All patients were administered injections in the operating room and subsequently moved to the recovery room. Depending on the circumstances, the patients either remained in the hospital or went home. The effect of CV-T therapy with AE was evaluated based on the percentage decrease in serum β-hCG level, calculated against the initial level obtained immediately before the first AE injection.

When the decrease rate of serum β-hCG level 2 h post-injection was > 20%, the procedure was deemed effective according to the protocol [11]. When the decrease rate was 13–20%, the effect was judged by measuring the serum β-HCG level again on the next day. If the decrease rate was < 13%, the effect was deemed insufficient, and a second AE dose was additionally injected and re-evaluated 2 h later.

For patients whose decline in β-hCG level was still < 20% on the next day, additional doses were administered until the required decline in β-hCG was achieved. From the second day onward, serum β-hCG level was checked every day for 3 days, then twice every alternate day, followed by twice every 5 days, and finally once a week, until the level reached < 0.3 mIU/mL. Patients who demonstrated a slow decline in β-hCG level were monitored for a longer period. In addition, the GS was monitored by TVU and, if necessary, by MRI until it disappeared completely.

### 2.3. Statistical Analysis

Descriptive statistics are reported as mean ± SD or number and percentage. 

## 3. Results

From April 2006 to December 2019, 242 patients with EP received CV-T therapy with AE. The mean (± SD) patient age was 38.1 ± 4.8 years. Of the 242 patients, 201 (83.1%) developed ectopic pregnancies after in vitro fertilization and embryo transfer (IVF-ET) treatment, and 41 (16.9%) developed the condition after spontaneous pregnancy; 103 (42.5%) were FHB-positive. The mean (range) values of gestational age (GA) and size of the gestational sac (GS) at the time of first injection in 242 patients were 6 w 4 d (range: 5 w 1 d–9 w 5 d) and 9.4 mm (range: 6–29 mm), respectively. The distribution of the number of patients based on the occurrence site of EP was as follows: fallopian tube pregnancy (200 patients; 82.6%), interstitial fallopian tube pregnancy (17 patients; 7.0%), CP (19 patients; 7.8%), CSSP (3 patients; 1.2%), and peritoneal pregnancy (3 patients; 1.2%) (Table 1).

**Figure 1:**
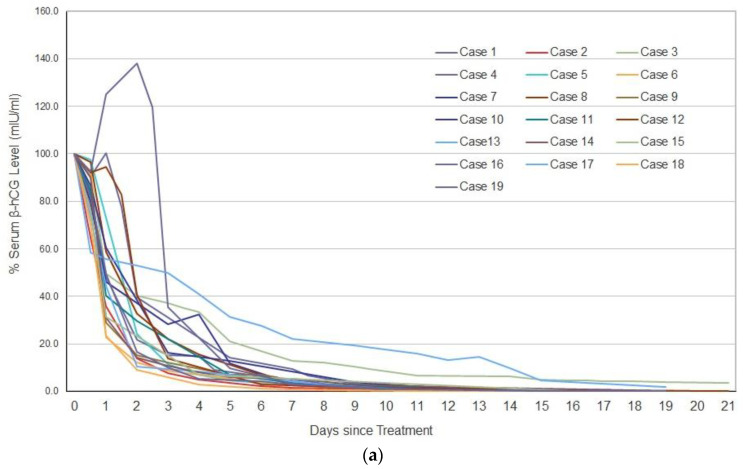

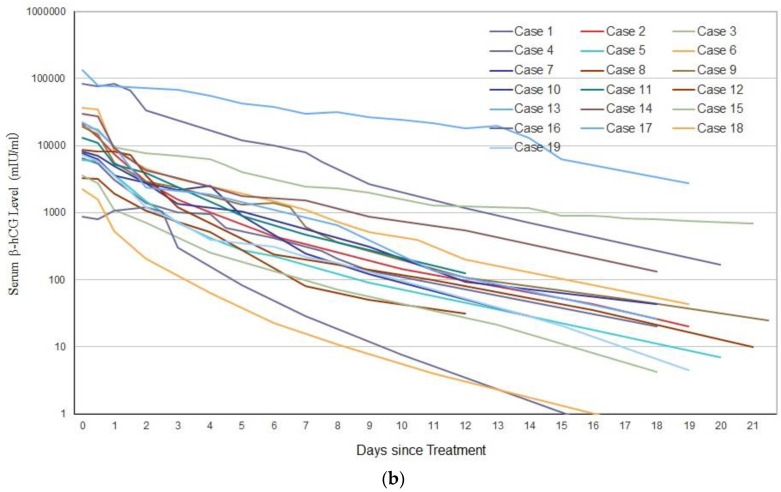
(**a**) Percentage change in the serum beta-human chorionic gonadotropin (β-hCG) level after chorionic villi-targeted therapy with absolute ethanol (CV-T therapy with AE) in 19 patients with cervical pregnancy. The *x*-axis shows days post-treatment; the *y*-axis shows the percentage of baseline serum β-hCG level. In all patients, the serum β-hCG level decreased to < 50% of the initial level within 3 days and to < 10% within 9–14 days. (**b**) Change in serum β-hCG level on a logarithmic scale. While the *x*-axis shows the number of days post-treatment, the *y*-axis shows the β-hCG level (mIU/mL). In all but one patient (with cervical pregnancy), the β-hCG level decreased to < 10 mIU/mL within 27 days of the treatment and to < 1.0 mIU/mL within 40 days.

**Figure 2:**
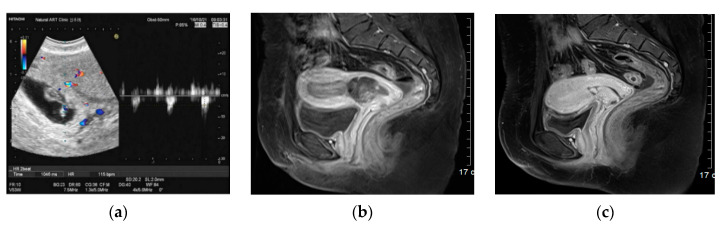
Transvaginal ultrasound Doppler image and pelvic MRI scan of cesarean section scar pregnancy with positive fetal heartbeat. (**a**) Transvaginal ultrasound Doppler image demonstrating FHB-positive CSSP. (**b**) Axial sections of pelvic MRI before the injection; initial serum β-hCG level was 43,596 mIU/mL and that at 2 h after the AE injection was 32,161 mIU/mL, demonstrating a reduction rate of 26.2%. (**c**) Axial sections of pelvic MRI on day 25 of the injection. Serum β-hCG level was 6.8 mIU/mL (on day 38, the level decreased to < 1.0 mIU/mL).

The mean (range) values of serum β-hCG level initially, and at 2 h after AE injection, for all patients, were 7034.6 mIU/mL (range: 347–135,040 mIU/mL) and 5691.5 mIU/mL (range: 313–78,890 mIU/mL), respectively. The mean (± SD) β-hCG percentage decrease was 17.4 ± 8.4% (95% CI: 15.6–19.2%) at 2 h after the injection. The average number of AE injections in 242 patients was 1.6 (range, 1–5), and the average dose of AE injections was 3.2 mL (range, 0.3–53.0 mL). Of the 222 patients who were successfully treated, 106 (47.7%) were able to complete the treatment with one AE injection while in the remaining 116 patients (52.3%), the serum β-HCG level either did not decrease enough or re-increased after the injection; therefore, AE was repeatedly injected. AE injection was performed twice in 76 patients (34.1%), thrice in 28 patients (12.6%), 4 times in 9 patients (4.0%), and 5 times in 3 patients (1.3%). In all but one patient with CP, the serum β-hCG level was reduced by 50% within 3 days, and up to < 10% of the initial level within 9–14 days. Serum β-hCG levels reached ≤ 10.0 mIU/mL within 30–36 days, and took only 40 days to reach < 1.0 mIU/mL. The condition of 26 patients (11.7%), whose serum β-HCG level had decreased but not reached a negative value (< 0.3 mIU/mL), was diagnosed as persistent trophoblastic disease. They were administered 7.5–10.0 mg MTX for 5 days in order to accelerate the reduction in β-hCG levels, and all completed the treatment successfully. The overall success rate of CV-T therapy with AE was 91.7% (222/242 patients), including 92.5% (185 patients) in fallopian tube pregnancy; 88.2% (15 patients) in interstitial fallopian tube pregnancy; 100% (19 patients) in CP (Figure 1); and 100% (3 patients) in CSSP (Figure 2). The success rate in treating FHB-positive patients (103 patients; 42.5%) reached 100% due to its clearly visible implantation site.

Twenty patients (8.2%) did not react to the therapy, since their β-hCG level did not decrease, and hence, laparoscopic surgery had to be performed to treat their conditions. The failure could be attributed to misdiagnosis of the implantation site by ultrasonography. Three patients with peritoneal pregnancies were observed to have a GS-like image with the white ring-like image near the ovary, and AE was injected into those sites. However, the treatment appeared to have no effect, and as there was no possible site of ectopic pregnancy found, laparoscopic operation was performed, which consequently revealed that the three patients had peritoneal ectopic pregnancies. One patient with CP showed an abnormal course of events. Her serum β-hCG level before the treatment was as high as 135,040 mIU/mL and, since the level continued to decrease poorly, she required four additional AE injections. Her initial AE dose was 17 mL and the serum β-hCG level, measured 2 h after the injection, was 78,890 mIU/mL (decreased by 41.6%). The total dose of four additional injections administered was 36 mL, which implied an overall dose of 53 mL. Although her serum β-hCG level decreased to < 50% within 3 days and < 10% within 14 days of the initial injection, a further decrease to < 10.0 mIU/mL and < 1.0 mIU/mL took 68 days and 190 days, respectively.

Of the 242 patients, 103 (42.5%) required 1% lidocaine^R^ (lidocaine hydrochloride monohydrate) or 0.5% Marcain^R^ (bupivacaine hydrochloride hydrate) as local anesthesia for the vaginal wall, whereas the remaining 139 patients (57.5%) received none. No other anesthesia/analgesia, including intravenous, inhaled, or oral medications, was used. Cephem antibiotics (Cefamezinα 2 g for injection; Astellas Pharma, Tokyo, Japan) were administered intravenously, once daily, 30 min prior to the procedure.

After the treatment, several patients tried to conceive again. As a result, 63 out of 145 patients (43.4%) with fallopian tube pregnancy became pregnant by IVF-ET and delivered babies; 7 out of 12 traceable patients (58.3%), who were treated for CP or CSSP, conceived, of which 4 delivered naturally while 3 delivered by cesarean section.

### Complications and Hospitalization

After the treatment, no rupture of the fallopian tubes, bleeding from the injection site, or infection, was observed. However, in 115 (57.5%) out of 200 patients with fallopian tube pregnancies, symptoms of peritoneal irritation were observed in the case where AE leaked into the abdominal cavity. For severe peritoneal irritation, Voltaren suppository 25 mg/50 mg (diclofenac sodium) or Sosegon tablets 25 mg (pentazocine hydrochloride) was administered. One patient with cervical pregnancy had a hematoma of approximately 2.5-cm diameter around the GS, and transvaginal ligation of the cervical arteries had to be performed to stop the persistent bleeding. Owing to this hematoma, the hyperechoic inner ring was difficult to identify by transvaginal ultrasonography (TVU), and a total of 10 mL of AE was administered over several injections. The β-hCG level dropped to < 0.2 mIU/mL within a month.

After the procedure, 48 (22.1%) out of 217 patients with tubal pregnancy and 10 (45.4%) out of 22 patients with CP or CSSP were hospitalized, in case of a need for emergency procedures due to responses such as rupture of the fallopian tube or uterine bleeding. The remaining 184 patients (76.0%) were treated on an outpatient basis only. The average length of hospital stay was 2.3 days (1–25 days), although one patient with CSSP, having a large lesion, required the longest hospital stay of 25 days.

## 4. Discussion

EP is a disease identified in women at an early stage of pregnancy, and is an absolute indication for surgery, since it is a serious risk factor for maternal mortality owing to acute abdomen [3,4]. In CP and CSSP, in particular, trophoblast cells may invade the cervical wall and cervical blood vessels, and interventions such as dilatation and curettage might be associated with a high risk of rupture, severe bleeding, and hemodynamic collapse [12]. The risk of bleeding associated with CSSP intervention is much higher than that in CP, since CSSP occurs in the cervical muscle layer of the cesarean section scar. Therefore, hysterectomy or uterine artery embolization has historically been the recommended primary treatment of these conditions [12,19,20,21].

In recent years, with the widespread use of ultrasonography and rapid diagnosis of serum β-hCG, unruptured and asymptomatic early EP is often detected, and options such as drug therapy may be considered. Protocols for non-surgical treatment of EP, including CP and CSSP, are based on the combination of local transabdominal or transvaginal injection of embryocides, including MTX [22], potassium chloride, and hyperosmolar glucose, into the GS, frequently with systemic administration of MTX and/or other embryocides [5,20,21,23,24]. Recently, promising results were reported in CSSP with the local injection of MTX alone [6]. MTX therapy has, thus, been recognized as a drug therapy for EP for nearly 40 years since the report by Tanaka et al. [22], and is now recommended as the first medical treatment option for EP diagnosed asymptomatically [7,8]. MTX therapy has also been confirmed to be an alternative to laparoscopic surgery [25].

However, the application of MTX is severely limited in its guidelines [7,8]. Since the level of β-HCG is stipulated to be between > 1500 and ≤ 5000 IU/mL, and cases with FHB are not considered, it can only be used for the treatment of early-stage pregnancy. In a recent retrospective study involving 101 women with tubal ectopic pregnancies, the cutoff β-hCG value, which determined successful methotrexate treatment in single administration, was found to be ≤ 1362 mIU/mL [26]. Furthermore, response to MTX occurs over 5 to 7 days, since MTX works metabolically by causing death of villi, subsequently leading to an initial decrease in the serum β-hCG level. This implies that even when MTX treatment is initiated, there is always a possibility of bleeding due to rupture of GS or hematoma at the implantation site, thus requiring a preparation for emergency surgery at all times under hospital management, causing stress to not only the patient herself but also the medical staff. It also requires a long period of time (two to three months) for the level of β-hCG to become negative [6], in addition to concerns over complications, including myelosuppression and digestive symptoms, such as mouth sores.

Based on our present study and previous studies [9,11], we found that the local injection of AE has advantages over conventional MTX-based local injection therapy in several respects. First, this method does not have the indication limitations, as described in the guidelines for MTX treatment, such as the presence or absence of FHB, or β-HCG levels. The characteristics of AE injection are: (1) AE causes a decrease in serum β-hCG level within just 2 h following the initial injection, which may reflect trophoblastic necrosis through dehydration and coagulation, immediately terminating the cellular function of chorionic villi. Therefore, it is effective, without exception, even in cases with high β-HCG or FHB. (2) Since AE injection takes only 2 h to manifest its effect, prognosis can be made at an early stage, enabling out-patient treatment. (3) In the event of an insufficient result, AE can be administered repeatedly, since it does not affect the hematopoietic functions, unlike MTX. (4) The decline in serum β-hCG level after the injection is rapid, producing a 50% decline within three days, and 90% decline in 9–14 days. It also becomes negative rapidly, in only 14–30 days. (5) Due to the pharmacological properties of AE, local hemostatic effect via microvascular thrombosis [27], as well as bactericidal effect, can be expected. (6) Finally, it is quite a low-cost treatment.

As a complication of AE injection, symptoms of peritoneal irritation may develop when AE leaks into the abdominal cavity. For that condition, an analgesic drug may be administered, although the condition is transient and usually resolves in 1–3 h.

**Figure 3:**
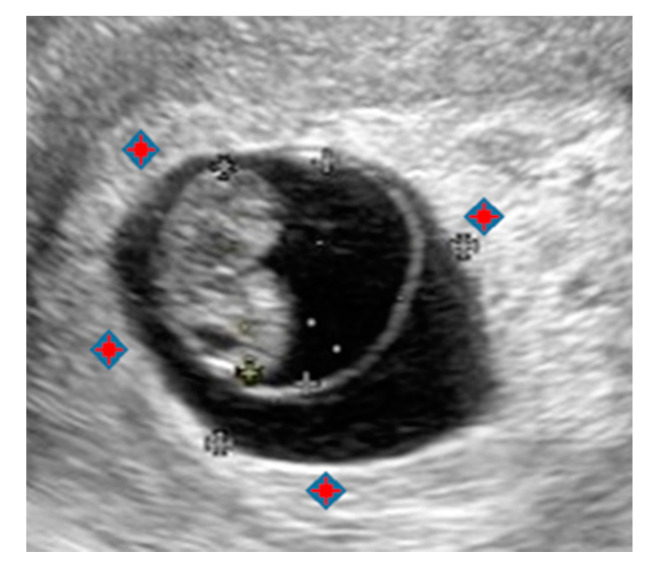
Ultrasonographic image at 7 w 3 d (19.3 × 13.8 mm). The red marks show the lacunar space where AE is injected.

AE was used for the induction of mid-trimester abortions as early as 1973 [28]. In 2006, we reported the trophoblast-targeted therapy for EP with local injection of AE into the GS [9]. The results, like in MTX, proved to be effective for early EPs up to 5 weeks of GA, although it had no effect on cases with high β-HCG or FHB [9]. Since then, we have worked on effective treatments for cases with high β-HCG and positive FHB. We focused on the phenomenon of the change in the drug’s effect from weakening to ineffective with the progression of pregnancy, consistent with the phenomenon of the shift in GS composition in early pregnancy from the chorionic sac to amniotic cavity, eventually forming the amniochorionic membrane [10,11]. This transition process, in which the chorionic cavity is overtaken by the amniotic cavity in the changing membrane structure of GS, can be observed with ultrasonography (Figure 3). From the end of 5–6 weeks of GA, the amniotic cavity begins to expand owing to an increase in amniotic fluid in the GS. At 7 weeks of GA, the amniotic cavity rapidly expands, while the chorionic cavity shrinks and disappears. At this time, the chorionic membrane is compressed against the wall to cover the outer surface of the amniotic cavity.

Finally, the amniotic membrane and chorionic membrane are fused together to form the strong amniochorionic membrane that acts as a barrier to trophoblasts and blocks the action of AE. The growing and thickening trophoblast cells do not allow AE to penetrate into the entire trophoblastic layer.

**Figure 4:**
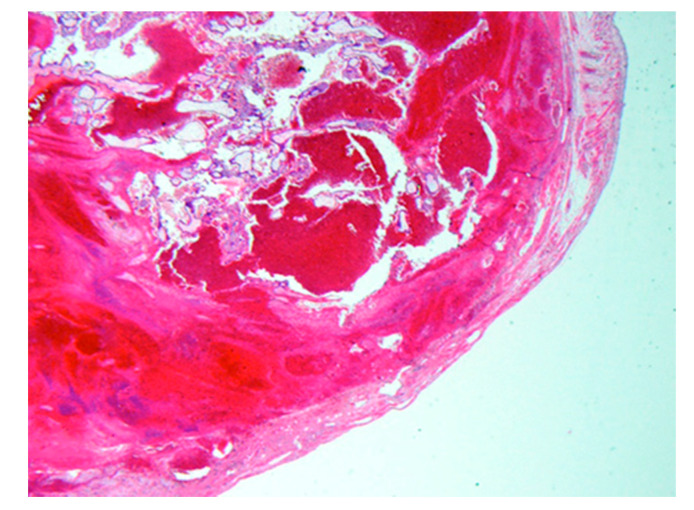
Histopathological examination of a persistent trophoblastic disease caused by insufficient AE effect on a patient with fallopian tube pregnancy (at 6 w 4 d; ×8). The fallopian tube was removed. Due to the effect of AE, protein degeneration was observed in most tissues, although live villous cells were observed in part.

Developing a method of local injection of AE that would not be affected by the strong barrier effect of amniochorionic membrane led to the “CV-T therapy with AE”. In contrast to the earlier method, in which AE was injected into GS after aspirating the liquid contained in the latter, CV-T therapy does not puncture GS, but rather injects AE locally into the lacunar space, where CV is present. It is a method of killing CV cells directly by the pharmacological action of AE (i.e., dehydration and protein denaturation) to stop physiological function and terminate the pregnancy (Figure 4).

**Figure 5:**
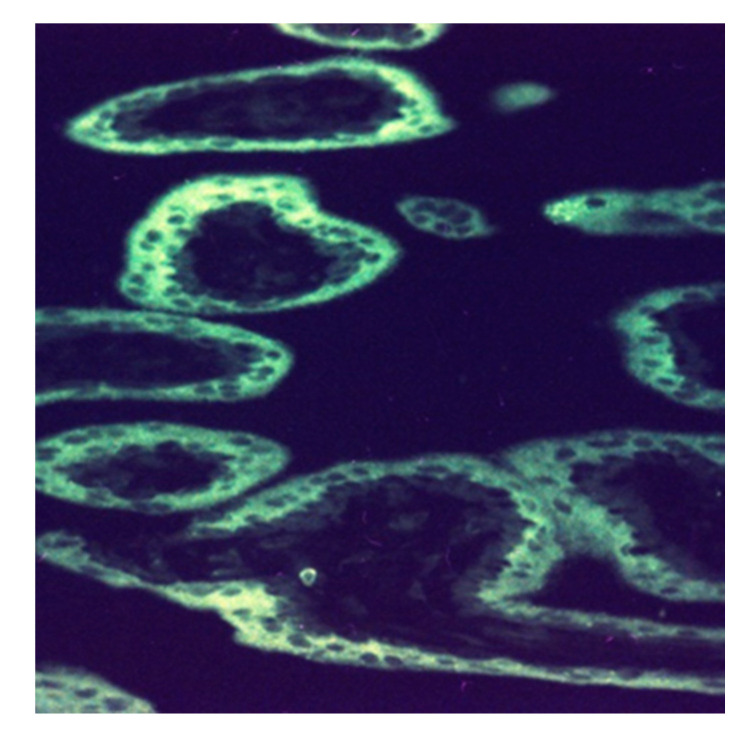
Human placental lactogen secretion from the syncytiotrophoblast at 12 w 2 d (×320). The fluorescent reaction with fluorescein-isothiocyanate (FITC)-labeled anti-hPL (rabbit) is confined to the syncytiotrophoblast layer of chorionic villi, thus creating the image of a “white ring”.

In the lacunar space, CV consisting of two layers of cells, differentiated from the trophoblast, projects in a dendritic shape toward the maternal side and makes contact points with the endometrium. The inner layer of CV is the actively proliferating cytotrophoblast, and the outer layer is formed from the syncytiotrophoblast, which erodes maternal tissues and anchors the villi to the endometrium. The syncytiotrophoblast plays the most important role in maintaining pregnancy by directly contacting the endometrium for gas exchange. It also secretes human placental lactogen (hPL) to absorb the nutrients, necessary for the fetus, from the mother (Figure 5); secretes hCG to maintain the corpus luteum graviditatis of the ovary; and regulates estrogen and progesterone secretion [29].

**Figure 6:**
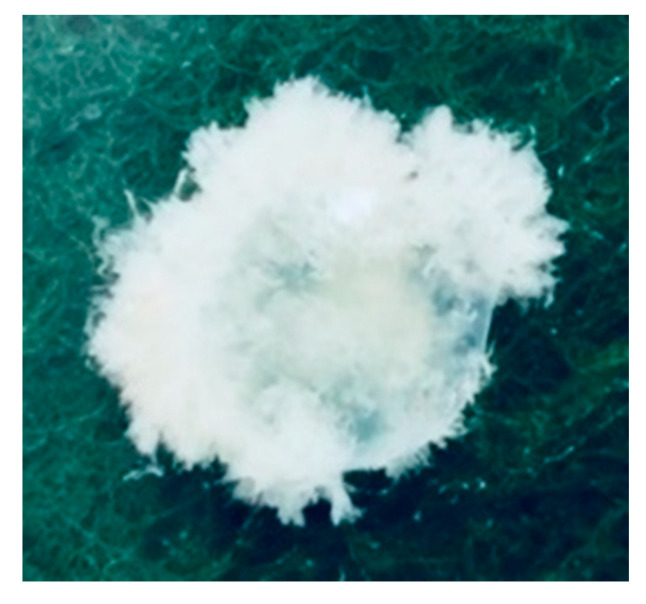
Chorionic villi at 7 w 5 d (30 × 27 mm).

The lacunar space cannot be observed directly; however, it is present in the decidua capsularis, thickened by the chorionic reaction around GS, and appears as a hyperechoic inner ring (white echogenic rim or “white ring”) on TVU (Figure 4) [18,19]. Figure 6 shows a photograph of the chorionic sac containing a fetus spontaneously aborted at 7 w 5 d. The chorionic villi regress and disappear, resulting in a smooth chorion (recognized from the 3 o’clock to 6 o’clock direction), or they persist and form a villous chorion (recognized from the 6 o’clock to 3 o’clock direction) to form a fetal component of the placenta.

At an early stage of pregnancy, CV exists ubiquitously over the entire surface of the chorionic sac and AE diffuses all around the space when injected into the lacunar space. However, CV is massed together in a part of the sac as pregnancy progresses to 7–8 weeks. Therefore, local injection of AE after 7–8 weeks of pregnancy should be targeted at a hyperechoic site. A transvaginal ultrasound Doppler image would be of help at this time point. The method of locally injecting the drugs has the advantage of a higher concentration of drug being delivered to the site with fewer side effects. Although success of the therapy depends on the skill of the physician, the puncturing technique has been drastically improved with the widespread use of IVF-ET, making room for the local injection method to be widely accepted.

Through this study, we found that the CV-T therapy with local injection of AE is a relatively simple procedure, effective for all types of patients with EP, including those with high βHCG or FHB. We also found it to be highly beneficial to both patients and physicians. However, since this is a retrospective study and there is no control group, a call for larger, multicenter trials would be appropriate for confirming the safety of this therapy.

## Figures and Tables

**Table 1:** Chorionic villi-targeted therapy with local injection of absolute ethanol for treatment of ectopic pregnancy conducted between April 2006 and December 2019.

Type of EP ^1^	No. of Cases (%)	FHB ^2^-Positive (%)	No. of Successful Cases (%)	Average AE ^3^ Dose (mL)	Average No. of AE Injections	No. of Failed Cases (%)
Tubal	200 (82.6)	85 (42.5)	185 (92.5)	2.9	1.6	15 (8.0)
Interstitial	17 (7.0)	12 (70.5)	15 (88.2)	3.6	1.9	2 (11.7)
CP ^4^	19 (7.8)	4 (21.0)	19 (100.0)	8.8	1.8	0
CSSP ^5^	3 (1.2)	2 (66.6)	3 (100.0)	15	2	0
Peritoneal	3 (1.2)	0	0	2.2	1	3 (100.0)
Total	242 (100.0)	103 (42.5)	222 (91.7)			20 (8.2)

^1^ Ectopic pregnancy; ^2^ fetal heartbeat; ^3^ absolute ethanol; ^4^ cervical pregnancy; ^5^ cesarean section scar pregnancy.

## Abbreviations

AE	Absolute ethanol
CP	Cervical pregnancy
CSSP	Cesarean section scar pregnancy
CV-T	Chorionic villi targeted
EP	Ectopic pregnancy
FHB	Fetal heartbeat
GA	Gestational age
GS	Gestational sac
IVF-ET	In vitro fertilization and embryo transfer
MRI	Magnetic resonance imaging
TVU	Transvaginal ultrasonography
